# Preventable Deaths During Widespread Community Hepatitis A Outbreaks — United States, 2016–2022

**DOI:** 10.15585/mmwr.mm7242a1

**Published:** 2023-10-20

**Authors:** Megan G. Hofmeister, Neil Gupta, Hepatitis A Mortality Investigators, Hepatitis A Mortality Investigators, Priscilla Lauro, E. Marilea Brock, Alan May, Cherie Smith, Olivia Arizmendi, Kerri Brown, Rachel H. Jervis, Ann Q. Shen, Ami P. Gandhi, Dawn Nims, Nicole Stone, Lauren Maxwell, Jennifer A. Khoury, Amanda K. Odegård, Douglas A. Thoroughman, Raychel N. Berkheimer, Jenna V. Iberg Johnson, Sean H. Simonson, Kompan Ngamsnga, Lindsay Bouton, Shauna Onofrey, Sharon Pagnano, Cole Burkholder, Theresa S. Kittle, John Bos, Kate Cleavinger, Nathan Koffarnus, Salena Savarda, Zuwen Qiu-Shultz, Devin Raman, Hannah Bowen, John J. Dreisig, Katrina E. Hansen, Patricia Amarilla, Troy Brancard, Mojisola Ojo, Marla M. Sievers, Justin P. Albertson, Susan M. Sullivan, Abdoulaye Diedhiou, LaKita D. Johnson, Jun Tang, Jane M. Brittingham, Danita C. Crear, Robb L. Garman, Elise M. Huebner, Binoj Peter, Marc Williamson, Bree Barbeau, MaryBeth DeMarco, Kelsey Holloman, Mary Chan, Hilary Armstrong, Jean-Jacques Kayembe Kashondo, Alana G. Hudson, Shannon McBee, Melissa A. Scott

**Affiliations:** 1 Division of Viral Hepatitis, National Center for HIV, Viral Hepatitis, STD, and TB Prevention, CDC.; Arizona Department of Health Services; Arkansas Department of Health; Arkansas Department of Health; Arkansas Department of Health; California Department of Public Health; Colorado Department of Public Health & Environment; Colorado Department of Public Health & Environment; Colorado Department of Public Health & Environment; Georgia Department of Public Health; Illinois Department of Public Health; Indiana Department of Health; Kansas Department of Health and Environment; Kentucky Department for Public Health; Kentucky Department for Public Health; CDC and Kentucky Department for Public Health; Louisiana Department of Health; Louisiana Department of Health; Louisiana Department of Health; Maryland Department of Health; Massachusetts Department of Public Health; Massachusetts Department of Public Health; Massachusetts Department of Public Health; Michigan Department of Health & Human Services; Mississippi State Department of Health; Missouri Department of Health and Senior Services; Missouri Department of Health and Senior Services; Missouri Department of Health and Senior Services; Nevada Department of Health and Human Services; Southern Nevada Health District; Southern Nevada Health District; New Hampshire Division of Public Health Services; New Hampshire Division of Public Health Services; New Hampshire Division of Public Health Services; New Jersey Department of Health; New Jersey Department of Health; New Jersey Department of Health; New Mexico Department of Health; North Carolina Department of Health and Human Services; North Carolina Department of Health and Human Services; South Carolina Department of Health and Environmental Control; South Carolina Department of Health and Environmental Control; South Carolina Department of Health and Environmental Control; Tennessee Department of Health; Tennessee Department of Health; Tennessee Department of Health; Texas Department of State Health Services; Texas Department of State Health Services; Dallas County Health and Human Services; Utah Department of Health and Human Services; Virginia Department of Health; Virginia Department of Health; Washington State Department of Health; , Public Health – Seattle & King County; Public Health – Seattle & King County; West Virginia Department of Health and Human Resources; West Virginia Department of Health and Human Resources; West Virginia Department of Health and Human Resources

**Summary**

**What is already known about this topic?**

Hepatitis A is a vaccine-preventable disease that typically causes mild, self-limited illness. Serious complications, including death, are rare, but are more frequent among older adults. Hepatitis A outbreaks associated with person-to-person transmission have been widespread in the United States since 2016.

**What is added by this report?**

During August 1, 2016–October 31, 2022, 27 U.S. states reported 315 hepatitis A outbreak–related deaths. Deaths peaked in 2019 and then decreased annually through 2022. Overall, 63% of decedents had at least one documented preexisting indication for hepatitis A vaccination.

**What are the implications for public health practice?**

Increased hepatitis A vaccination coverage, particularly among adults at increased risk for infection with hepatitis A virus or for severe disease from infection, is critical to preventing future hepatitis A deaths.

## Abstract

Hepatitis A is acquired through the fecal-oral route and is preventable by a safe and effective vaccine. Although hepatitis A is generally mild and self-limited, serious complications, including death, can occur. Since 2016, widespread hepatitis A outbreaks have been reported in 37 U.S. states, primarily among persons who use drugs and those experiencing homelessness. Nearly twice as many hepatitis A–related deaths were reported during 2016–2022 compared with 2009–2015. CDC analyzed data from 27 hepatitis A outbreak-affected states* that contributed data during August 1, 2016–October 31, 2022, to characterize demographic, risk factor, clinical, and cause-of-death data among 315 outbreak-related hepatitis A deaths from those states. Hepatitis A was documented as an underlying or contributing cause of death on 60% of available death certificates. Outbreak-related deaths peaked in 2019, and then decreased annually through 2022. The median age at death was 55 years; most deaths occurred among males (73%) and non-Hispanic White persons (84%). Nearly two thirds (63%) of decedents had at least one documented indication for hepatitis A vaccination, including drug use (41%), homelessness (16%), or coinfection with hepatitis B (12%) or hepatitis C (31%); only 12 (4%) had evidence of previous hepatitis A vaccination. Increasing vaccination coverage among adults at increased risk for infection with hepatitis A virus or for severe disease from infection is critical to preventing future hepatitis A–related deaths.

## Introduction

Hepatitis A virus (HAV) infections are acquired through fecal-oral transmission. Although hepatitis A is generally mild and self-limited, serious complications, including death, can occur (*1*,*2*). Hepatitis A is preventable by a highly effective and safe vaccine (*3*). Since 2016, hepatitis A outbreaks associated with person-to-person transmission have been reported in 37 states, involving approximately 44,900 cases, 27,450 hospitalizations, and 423 deaths as of October 6, 2023 (*4*,*5*). These outbreaks have disproportionately affected persons who use drugs and persons experiencing homelessness, who might be at increased risk for HAV infection because of poor hygiene practices, lack of access to sanitation, or crowded living conditions (*3*). Nearly twice as many deaths involving hepatitis A in the United States occurred during 2016–2022 compared with 2009–2015 (*6*).

## Methods

Deidentified demographic, risk factor, and clinical data from state outbreak databases, along with place and cause of death data from death certificates, were requested for all hepatitis A outbreak–related deaths from the 32 state health departments that publicly reported at least one outbreak-related death during August 1, 2016–October 31, 2022. All hepatitis A cases met the Council of State and Territorial Epidemiologists’ hepatitis A surveillance case definition (*7*). Risk factors were self-reported during the exposure period (15–50 days before symptom onset). Outbreak-related deaths were defined as deaths that state health departments determined were attributable to hepatitis A. Death certificate data were reviewed to determine if hepatitis A was listed as 1) a cause of death (listed anywhere in the chain of events that directly caused death) or 2) a significant condition contributing to death. The analysis was conducted using SAS software (version 9.4; SAS Institute). This activity was reviewed by CDC, deemed not research, and conducted consistent with applicable federal law and CDC policy.^†^

## Results

CDC analyzed data from 27 (84%) states contributing data, among the 32 outbreak-affected states that had publicly reported at least one hepatitis A outbreak-related death. These 27 states accounted for 315 outbreak-related deaths, approximately 75% of publicly reported hepatitis A outbreak-related deaths (approximately 71% of publicly reported hepatitis A outbreak-related cases) at the time of the request for data. Deaths occurred during September 13, 2016–June 20, 2022 (Figure), most among males (73%) and non-Hispanic White persons (84%); the median age at death was 55 years (Table 1). Outbreak-related deaths peaked in 2019, and then decreased annually through 2022. The median interval between symptom onset and date of death was 17 days. Among decedents, 91% were hospitalized, 77% had jaundice, and one (<1%) underwent liver transplantation; among the 218 hospitalized decedents with available information, the median length of hospitalization was 7 days (IQR = 4–14 days). Drug use was the most commonly reported risk factor for HAV infection (41%), followed by homelessness or unstable housing (16%). Coinfection with hepatitis C (31%) was more prevalent than was coinfection with hepatitis B (12%). Only 12 decedents (4%) had evidence of previous hepatitis A vaccination; 63% had at least one documented indication for vaccination according to recommendations of the Advisory Committee on Immunization Practices (*3*).

**FIGURE:**
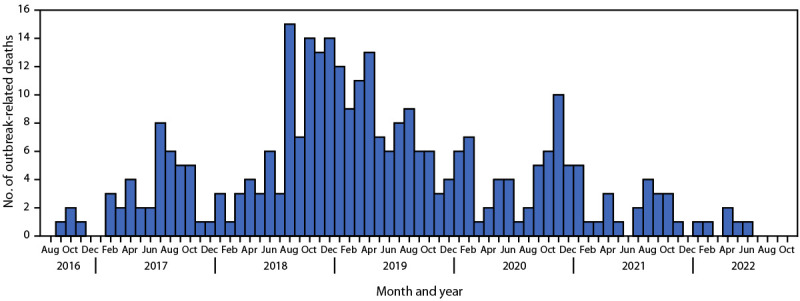
Date of hepatitis A outbreak–related deaths* ― 27 U.S. states, August 1, 2016–October 31, 2022 * Among 306 outbreak-related deaths for which the date of death was known.

**TABLE 1:** Characteristics of persons whose death was related to a hepatitis A outbreak — 27 U.S. states,* August 1, 2016–October 31, 2022^†^

Characteristic (no. with available data)	No. (%)
**Total no. of reported hepatitis A outbreak–related deaths**	**315**
**Death date range (306)**	Sep 13, 2016–Jun 20, 2022
**Year of death (306)**	
2016	4 (1.3)
2017	39 (12.7)
2018	86 (28.1)
2019	94 (30.7)
2020	53 (17.3)
2021	24 (7.8)
2022	6 (2.0)
**Interval between symptom onset and death, days, median (IQR) (306)^§^**	17.0 (9.0–33.0)
**Sex**	
Female	85 (27.0)
Male	230 (73.0)
**Age group at death, yrs (314)**	
0–19	0 (—)
20–29	12 (3.8)
30–39	27 (8.6)
40–49	62 (19.8)
50–59	97 (30.9)
≥60	116 (36.9)
**Median age at death, yrs (range) (314)**	55 (24–96)
**Race and ethnicity**	
American Indian or Alaska Native, non-Hispanic	3 (1.0)
Black or African American, non-Hispanic	15 (4.8)
White, non-Hispanic	265 (84.1)
Hispanic or Latino	13 (4.1)
Multiple races, non-Hispanic	2 (0.6)
Unknown	17 (5.4)
**Jaundice or scleral icterus**	
Yes	242 (76.8)
No	52 (16.5)
Unknown	21 (6.7)
**Hospitalized**	
Yes	288 (91.4)
Length of hospitalization, days, median (IQR) (218)	7.0 (4.0–14.0)
No	24 (7.6)
Unknown	3 (1.0)
**Liver transplant**	
Yes	1 (0.3)
No	134 (42.5)
Unknown	180 (57.1)
**Hepatitis A vaccination status**	
Ever vaccinated (≥1 dose)^¶^	12 (3.8)
Unvaccinated	146 (46.4)
Unknown	157 (49.8)
Risk factors**	
Any drug use	
Yes	128 (40.6)
No	113 (35.9)
Unknown	74 (23.5)
Injection drug use	
Yes	76 (24.1)
No	140 (44.4)
Unknown	99 (31.4)
Noninjection drug use	
Yes	77 (24.4)
No	123 (39.1)
Unknown	115 (36.5)
Experiencing homelessness or unstable housing	
Yes	50 (15.9)
No	217 (68.9)
Unknown	48 (15.2)
Male-to-male sexual contact (230)^††^	
Yes	7 (3.0)
No	91 (39.6)
Unknown	132 (57.4)
International travel	
Yes	2 (0.6)
No	200 (63.5)
Unknown	113 (35.9)
Incarcerated	
Yes	9 (2.9)
No	160 (50.8)
Unknown	146 (46.4)
Epidemiologically linked^§§^	
Yes	30 (9.5)
No	99 (31.4)
Unknown	186 (59.1)
**Coinfection**	
Hepatitis B	
Yes	37 (11.7)
No	231 (73.3)
Unknown	47 (14.9)
Hepatitis C	
Yes	97 (30.8)
No	179 (56.8)
Unknown	39 (12.4)
HIV	
Yes	0 (—)
No	140 (44.4)
Unknown	175 (55.6)

* The following 27 states contributed data for the analysis: Arizona, Arkansas, California, Colorado, Georgia, Illinois, Indiana, Kansas, Kentucky, Louisiana, Maryland, Massachusetts, Michigan, Mississippi, Missouri, Nevada, New Hampshire, New Jersey, New Mexico, North Carolina, South Carolina, Tennessee, Texas, Utah, Virginia, Washington, and West Virginia.

^†^ Missing data were categorized as unknown for the purpose of the analysis.

^§^ When symptom onset date was not available, date of collection of the specimen that tested positive for hepatitis A virus immunoglobulin M antibodies was used as a proxy for symptom onset date.

^¶^ Characteristics related to hepatitis A vaccination status of those reported as “ever vaccinated” were not available (the number of hepatitis A vaccine doses received, whether the hepatitis A vaccine doses were self-reported or confirmed in a state immunization information system, and the timing of doses). These do not necessarily represent instances of hepatitis A vaccine failure (e.g., they could represent instances of postexposure prophylaxis having been administered outside the recommended 14-day window when it is effective).

** Risk factors were ascertained during the exposure period (15–50 days before symptom onset).

^††^ Restricted to males.

^§§^ Contact (e.g., household or sexual) with a laboratory-confirmed hepatitis A case 15–50 days before symptom onset.

Death certificate data were provided by 25 (93%) of 27 states for 272 (86%) decedents (Table 2). Hepatitis A was not listed on 108 (40%) of the death certificates. Among the 164 (60%) death certificates with hepatitis A documented, hepatitis A was listed as a cause of death on 142 (87%) and as a significant condition contributing to death on 26 (16%).

**TABLE 2:** Death certificate analysis of hepatitis A outbreak–related deaths — 25 U.S. states,* August 1, 2016–October 31, 2022

Characteristic (no. with available data)	No. (%)
**Death certificate available (315)^†^**	
Yes	272 (86.3)
No	22 (7.0)
Unknown	21 (6.7)
**Hepatitis A status when death certificate was available (272)^§^**	
Hepatitis A not listed on death certificate	108 (39.7)
Hepatitis A listed on death certificate	164 (60.3)
Hepatitis A listed as a cause of death (164)^¶^	142 (86.6)
Hepatitis A listed as a significant condition contributing to death (164)^¶^	26 (15.9)
**Place of death (272)^§^**	
Inpatient facility	226 (83.1)
Hospice facility	16 (5.9)
Decedent’s home	13 (4.8)
Other	8 (2.9)
Emergency department or outpatient facility	6 (2.2)
Nursing home or long-term care facility	3 (1.1)

* The following 25 states contributed death certificate data for the analysis: Arizona, Arkansas, California, Colorado, Georgia, Illinois, Indiana, Kansas, Kentucky, Louisiana, Massachusetts, Michigan, Mississippi, Missouri, Nevada, New Hampshire, New Jersey, New Mexico, North Carolina, South Carolina, Tennessee, Texas, Utah, Washington (Seattle-King County only), and West Virginia.

^†^ Calculated among all 315 hepatitis A outbreak–related deaths from 27 states.

^§^ Calculated among 272 hepatitis A outbreak–related decedents from 25 states for whom death certificate data was available.

^¶^ Categories are not mutually exclusive.

## Discussion

Data from 27 states were analyzed to characterize the epidemiology of 315 hepatitis A outbreak–related deaths during August 1, 2016–October 31, 2022. Deaths occurred predominantly among males, non-Hispanic White persons, and persons aged ≥50 years. Nearly two thirds of decedents had at least one documented indication for hepatitis A vaccination, including drug use, homelessness, or coinfection with hepatitis B virus or hepatitis C virus; however, only 12 decedents had evidence of previous hepatitis A vaccination, indicating substantial missed opportunities to prevent hepatitis A deaths. Lack of stable housing and substance use disorder are commonly associated with viral hepatitides (*3*,*4*) and interact to increase disease incidence and health disparities. Although hepatitis A is usually a self-limited and preventable disease, it can have lethal consequences when introduced into populations with limited access to preventive care, unstable housing situations, inadequate access to sanitary services, or coexisting liver disease. These findings underscore the importance of integrated, comprehensive services, including vaccination, harm reduction, substance use disorder treatment, and hygiene and sanitation, to improve the health of medically underserved populations.

Among 272 outbreak-related decedents with available death certificate data, hepatitis A was listed as a cause of death or significant condition contributing to death on only 60% of death certificates, suggesting a substantial underestimation of hepatitis A mortality related to the outbreaks associated with person-to-person transmission in U.S. national vital statistics data. The 60% reporting rate for hepatitis A outbreak–related deaths is substantially higher than reporting rates for hepatitis B and hepatitis C; in previous death certificate analyses of cohorts of patients with chronic hepatitis B and chronic hepatitis C, only 19% of decedents had hepatitis B or hepatitis C reported on their death certificates (*8*,*9*).

### Limitations

The findings in this report are subject to at least five limitations. First, states did not use a standardized hepatitis A–related death definition, which might have resulted in differential classification of deaths as being related to hepatitis A. Second, death from hepatitis A is not a reportable condition and health departments might not have identified all outbreak-related hepatitis A deaths. Third, risk factor data were self-reported and subject to social desirability and recall biases and missingness. Consequently, information about additional decedents with indications for hepatitis A vaccination was unavailable. Fourth, vaccination information was missing for nearly one half of decedents; however, HAV infection after vaccination or appropriately timed postexposure prophylaxis is rare given the documented high immunogenicity of the vaccine (*3*). Finally, although the analysis captured nearly three quarters of publicly reported outbreak-related deaths, the results might not be generalizable to all outbreak-related deaths in the United States.

### Implications for Public Health Practice

Hepatitis A is a vaccine-preventable disease; safe and highly effective vaccines have been available for decades (*3*). Substantial progress has been made in controlling the recent outbreaks through intensive efforts by health departments, including outreach through mobile vans and foot teams, nontraditional vaccination clinics in jails and homeless shelters, and partnerships with sheriffs’ associations and other community-based partners to expand vaccination coverage. As of October 2023, 34 states have declared ends to their outbreaks; however, many susceptible adults, particularly among persons who use drugs, persons experiencing homelessness, and persons with chronic liver disease, remain at increased risk for HAV infection or severe disease from HAV infection (*5*,*10*). Increased hepatitis A vaccination coverage is critical to maintain the progress that has been made and prevent future hepatitis A deaths.

## Footnotes

* The following 27 states contributed data for the analysis: Arizona, Arkansas, California, Colorado, Georgia, Illinois, Indiana, Kansas, Kentucky, Louisiana, Maryland, Massachusetts, Michigan, Mississippi, Missouri, Nevada, New Hampshire, New Jersey, New Mexico, North Carolina, South Carolina, Tennessee, Texas, Utah, Virginia, Washington, and West Virginia.

† 45 C.F.R. part 46, 21 C.F.R. part 56; 42 U.S.C. Sect. 241(d); 5 U.S.C. Sect. 552a; 44 U.S.C. Sect. 3501 et seq.
